# Single nucleotide polymorphisms in bone turnover-related genes in Koreans: ethnic differences in linkage disequilibrium and haplotype

**DOI:** 10.1186/1471-2350-8-70

**Published:** 2007-11-26

**Authors:** Kyung-Seon Kim, Ghi-Su Kim, Joo-Yeon Hwang, Hye-Ja Lee, Mi-Hyun Park, Kwang-joong Kim, Jongsun Jung, Hyo-Soung Cha, Hyoung Doo Shin, Jong-Ho Kang, Eui Kyun Park, Tae-Ho Kim, Jung-Min Hong, Jung-Min Koh, Bermseok Oh, Kuchan Kimm, Shin-Yoon Kim, Jong-Young Lee

**Affiliations:** 1 Center for Genome Science, National Institute of Health, 5 Nokbun-dong, Eunpyung-gu, Seoul 122-701, Republic of Korea; 2 Skeletal Diseases Genome Research Center, Kyungpook National University Hospital, 44-2, Samduk 2-ga, Jung-gu, Daegu, 700-412, Republic of Korea; 3 Division of Endocrinology and Metabolism, University of Ulsan College of Medicine, Asan Medical Center, Seoul, 138-736, Republic of Korea; 4 Department of Genetic Epidemiology, SNP Genetics, Inc., Rm 1407, 14^th ^floor, B-dong, WooLim Lion's Valley, 371-28, Gasan-dong, Geumcheon-gu, Seoul, 153-803, Republic of Korea; 5 World Meridian Venture Center 10F, #60-24, Gasan-dong, Geumcheon-gu, Seoul 153-023, Republic of Korea; 6 Department of Pathology and Regenerative Medicine, School of Dentistry, Kyungpook National University, 188-1 Samduk 2-ga, Jung-gu, Daegu, 700-412, Republic of Korea; 7 Department of Orthopedic Surgery, Kyungpook National University School of Medicine, 50, Samduk 2-ga, Jung-gu, Daegu, 700-412, Republic of Korea

## Abstract

**Background:**

Osteoporosis is defined as the loss of bone mineral density that leads to bone fragility with aging. Population-based case-control studies have identified polymorphisms in many candidate genes that have been associated with bone mass maintenance or osteoporotic fracture. To investigate single nucleotide polymorphisms (SNPs) that are associated with osteoporosis, we examined the genetic variation among Koreans by analyzing 81 genes according to their function in bone formation and resorption during bone remodeling.

**Methods:**

We resequenced all the exons, splice junctions and promoter regions of candidate osteoporosis genes using 24 unrelated Korean individuals. Using the common SNPs from our study and the HapMap database, a statistical analysis of deviation in heterozygosity depicted.

**Results:**

We identified 942 variants, including 888 SNPs, 43 insertion/deletion polymorphisms, and 11 microsatellite markers. Of the SNPs, 557 (63%) had been previously identified and 331 (37%) were newly discovered in the Korean population. When compared SNPs in the Korean population with those in HapMap database, 1% (or less) of SNPs in the Japanese and Chinese subpopulations and 20% of those in Caucasian and African subpopulations were significantly differentiated from the Hardy-Weinberg expectations. In addition, an analysis of the genetic diversity showed that there were no significant differences among Korean, Han Chinese and Japanese populations, but African and Caucasian populations were significantly differentiated in selected genes. Nevertheless, in the detailed analysis of genetic properties, the LD and Haplotype block patterns among the five sub-populations were substantially different from one another.

**Conclusion:**

Through the resequencing of 81 osteoporosis candidate genes, 118 unknown SNPs with a minor allele frequency (MAF) > 0.05 were discovered in the Korean population. In addition, using the common SNPs between our study and HapMap, an analysis of genetic diversity and deviation in heterozygosity was performed and the polymorphisms of the above genes among the five populations were substantially differentiated from one another. Further studies of osteoporosis could utilize the polymorphisms identified in our data since they may have important implications for the selection of highly informative SNPs for future association studies.

## Background

Bone is continuously remodeled in vertebrates through coordinated phases of bone formation and resorption in order to maintain bone volume and phosphorus and calcium homeostasis [1]. Bone remodeling by direct contact with bone cells or by the release of soluble effectors is also altered by other cell protagonists present in the bone microenvironment such as monocytes/macrophages, lymphocytes, and endothelial cells [2]. In the disease state, the loss of bone homeostasis is potentially associated with changes in the numerous cellular protagonists that are responsible for the interactions between bone tissue, the immune system, and the vascular compartment. The study of bone homeostasis can therefore be utilized to elicit a better understanding of the pathologies associated with bone diseases such as osteoporosis [2]. Bone mass also has a very strong genetic determination: Twin and family studies showed that genetic factor could cause 50 to 90% of variance in bone mineral density (BMD) [3-8]. In addition, both the calcium-sensing receptor (*CASR*) and the interleukin 6 (*IL-6*) are important candidate genes for osteoporosis as well as in bone and mineral metabolism. These genes may have effects on BMD variation in Chinese nuclear families [9]. Determining SNPs for bone remodeling-related genes is becoming a more feasible and efficient tool for analyzing the processes associated with osteoporosis. However, an investigation of the distribution of SNPs within human populations is laborious and costly, mainly due to the necessity of testing large numbers of individuals and SNPs. Some SNPs for bone remodeling genes have already been reported; however, there are significant differences in allele frequency distributions among population groups, indicating that the populations exhibit genetic heterogeneity with respect to the incidence of these SNPs. Moreover, racial differences in the prevalence of certain alleles could account for a certain proportion of bone disease trait variation between different ethnicities [10]. The genetic variability of Asian and Caucasian populations was observed at restriction sites exhibiting polymorphisms of five important candidate genes for BMD: *CASR*-*BsaH*I, alpha 2HS-glycoprotein (*AHSG*)-*Sac*I, estrogen receptor alpha (*ESR1*)-*Pvu*II and *Xba*I, vitamin D receptor (*VDR*)-*Apa*I and parathyroid hormone (*PTH*)-*BstB*I. The results of the statistical analysis between the two populations revealed a significant allelic and genotypic differentiation in polymorphisms associated with osteoporosis. Intra- and inter-population variability implies that the studied pattern of variation at some loci may be affected by various types of natural selection [11]. A case-control approach is normally used to investigate the association of osteoporosis with SNPs in osteoporosis-related genes. A few of the newly discovered candidate genes (*PLXNA2, CAT *and *SEMA7A*) in our study were also used in case-control association studies in a Korean population [12-14]. These genes were screened in 24 individuals and then were genotyped in 560 postmenopausal women to compare gene and bone properties. Statistical analyses found a genetic linkage of the SNPs and haplotypes from the above genes with a risk of vertebral fracture or with BMD at the lumbar spine and at the femur neck [12-14]. Thus, to facilitate further association studies using SNPs of genes involved in osteoporosis, we selected 81 candidate genes involved in bone formation and resorption. We have characterized the genetic variants of these candidate osteoporosis genes, including gene-based haplotype diversity. These SNPs may be useful for genetic association studies that compare the SNP and haplotype information of ethnic groups.

## Methods

### Subjects and candidate genes

The study population consists of 24 unrelated Korean individuals, 11 men and 13 women, who were recruited from Ansan and Ansung area. The men were aged between 41 and 65 years (mean ± SD: 57.8 ± 8.5 years) and the women were aged between 41 and 62 years (mean ± SD: 52.6 ± 6.9 years). They were used for SNP screening and immortalized B lymphocyte cell line generation (cell line IDs GRB2015717, GRB2014744, GRB2014719, GRB2014754, GRB2015301, GRB2014712, GRB2012585, GRB2012949, GRB2012816, GRB2013123, GRB2012811, GRB2012998, GRB2015263, GRB2014890, GRB2014112, GRB2014896, GRB2014197, GRB2010947, GRB2021291, GRB2021404, GRB2021105, GRB2022466, GRB2026940, GRB2021302). Informed consent was obtained from all of the subjects, and this study was approved by the Institutional Review Board of the Korea National Institute of Health. Candidate osteoporosis genes were selected based on their function in bone/chondrocyte formation or bone resorption according to reports in the literature. We included the following genes of interest: those that promote or inhibit bone/chondrocyte formation; those that promote or inhibit bone resorption; and those involved in adipocyte differentiation. Genes of interest that promote bone/chondrocyte formation are as follows: *FGFs *[15], *SOX5,6,9 *[15], *BMPs *[16], *LGALS3 *[17], *LGALS1 *[18], *DLX5 *[16,19], *MSX2 *[19], *SP7 *[19], *CBFB *[20], *TGFBI *[21], *MSX1 *[22], *BGLAP *[23], *SPP1 *[24], *IBSP *[24], *IL1RN *[25], *CTNNB1 *[16,26], *WNTs *[26], *TCF4 *[27], *OMD *[28], *VEGFs *[29], *DMP1 *[30], *IL13 *[31], *AR *[32], *CYP17A1 *[32,33] and *CYP19A1 *[32,33]. Genes of interest that inhibit bone/chondrocyte formation are as follows: *PTHrP/PTHR1 *[15,19], *NPY2R *[19], *PPARG *[19], *TWIST1 *[16,24], *DKK1 *[26], *PTH *[19,34], *AHSG *[35], *PPP3CA *[36], *WIF1 *[37], *MEPE *[38] and *IL10 *[39,40]. Genes of interest that promote bone resorption are as follows: *PTHrP/PTHR1 *[15,19], *PTH *[19,34], *PTGS2 *[26], *IL4 *[34], *IL6ST *[41], *CTSK *[42], *H+ATPase *[42], *ITGA1 *[42,43], *NFKB *[42,44], *CALCR *[44], *CLCN7 *[44], *FOS *[44,45], *FOSB *[42,45], *FOSL2 *[46,47], *ITGAV *[42,44], *CSK *[42], *TRAF6 *[42,44], *MITF *[44], *CCR1 *[48], *NFATC1 *[49], *JDP2 *[50], *IL15 *[51], *PTK2B *[52], *CASR *[53], *SEMA7A *[54], *PTGER4 *[55] and *PLXNA2 *[12]. Genes of interest that inhibit bone resorption are as follows: *IL13 *[31], *AR *[32], *IL3 *[56], *ZNF675 *[57], *GPX1 *[58] and *CAT *[59]. In additional, a decrease in bone volume that occurs with age and in osteoporosis is accompanied by an increase in adipose tissue in the bone marrow, suggesting a dysregulation of the mesenchymal stem cell differentiation pathway in favour of adipogenesis. Therefore, we also included the following adipocyte differentiation genes: *PPARs *[19], *CEBPB *[19,47] and *DBI *[60].

### Resequencing analysis

To identify SNPs in the 81 candidate osteoporosis genes (Table 1), we resequenced all exons, including the coding region, the 5' UTR and the 3' UTR up to the splice junctions, as well as the promoter regions of approximately 0.5 kb proximal to the transcription start site in genomic DNA samples. For sequencing analysis, genomic DNA information was obtained from GenBank. Polymerase chain reaction (PCR) primers were designed using the Primer 3 program [61]. Genomic DNA was isolated from the 24 immortalized B lymphocyte cell lines of the selected subjects. PCR products were sequenced using the BigDye Terminator v3.1 cycle sequencing kit (Applied Biosystems, Foster City, CA) and an ABI 3730 automated sequencer (Applied Biosystems). SNPs were detected by multiple alignments of the sequences using the Phred/Phrap/Consed package [62,63] and polyphred [64]. All data for the SNPs discovered in the Korean samples have been deposited in the KSNP database [65].

**Table 1:** Gene information for candidate osteoporosis genes

**Gene Symbol**	**Gene Name**	**Locus ID**	**NM_#**	**Genomic Size**	**Exon #**
BGLAP	Bone gamma-carboxyglutamate (gla) protein (osteocalcin)	1q25-q31	NM_000711	263037	8
CALCR	Calcitonin receptor	7q21.3	NM_001742	149952	13
IL6ST	Interleukin 6 signal transducer (gp130, oncostatin M receptor)	5q11	NM_002184	54069	17
LGALS3	Lectin, galactoside-binding, soluble, 3 (galectin 3)	14q21-q22	NM_002306	16124	6
PPARG	Peroxisome proliferative activated receptor, gamma	3p25	NM_005037	146417	7
PTH	Parathyroid hormone	11p15.3-p15.1	NM_000315	3966	3
SP7	Sp7 transcription factor(osterix)	12q13.13	NM_152860	9176	2
TGFBI	Transforming growth factor, beta-induced, 68 kDa	5q31	NM_000358	34810	17
AR	Androgen receptor	Xq11.2-q12	NM_000044	180246	8
BMP7	Bone morphogenetic protein 7	20q13	NM_001719	95747	7
AHSG	Alpha 2 HS-glycoprotein	3q27	NM_001622	8219	7
BMP2	Bone morphogenetic protein 2	20p12	NM_001200	10563	3
BMP4	Bone morphogenetic protein 4	14q22-q23	NM_001202	7156	4
BMP6	Bone morphogenetic protein 6	6p24-p23	NM_001718	154718	7
CBFB	Core-binding factor, beta subunit	16q22.1	NM_022845	71907	6
CTSK	Cathepsin K	1q21	NM_000396	12126	8
DLX5	Distal-less homeobox 5	7q22	NM_005221	4436	3
IBSP	Integrin-binding sialoprotein (bone sialoprotein, bone sialoprotein II)	4q21-q25	NM_004967	12373	7
IL1RN	Interleukin 1 receptor antagonist	2q14.2	NM_000577	16123	5
LGALS1	Lectin, galactoside-binding, soluble, 1 (galectin 1)	22q13.1	NM_002305	4165	4
MSX1	Msh homeobox homolog 1	4p16.3-p16.1	NM_002448	4053	3
MSX2	Msh homeobox homolog 2	5q34-q35	NM_002449	6300	2
PTHLH	Parathyroid hormone-like hormone	12p12.1-p11.2	NM_002820	9663	4
PTHR1	Parathyroid hormone receptor 1	3p22-p21.1	NM_000316	26052	16
RUNX1	Runt-related transcription factor 1	21q22.3	NM_001754	261497	8
SPP1	Secreted phosphoprotein 1 (osteopontin, bone sialoprotein I)	4q21-q25	NM_000582	7761	6
TWIST1	Twist homolog 1 (acrocephalosyndactyly 3; Saethre-Chotzen syndrome)	7p21.2	NM_000474	2203	2
CEBPB	CCAAT/enhancer binding protein (C/EBP), beta	20q13.1	NM_005194	1837	1
CYP17A1	Cytochrome P450, family 17, subfamily A, polypeptide 1	10q24.3	NM_000102	6885	8
CYP19A1	Cytochrome P450, family 19, subfamily A, polypeptide 1	15q21.1	NM_000103	129125	10
IL10	Interleukin 10	1q31-q32	NM_000572	4892	5
IL4	Interleukin 4	5q31.1	NM_000589	5996	4
NFKB1	Nuclear factor of kappa light polypeptide gene enhancer in B-cells 1 (p105)	4q24	NM_003998	115989	24
VEGF	Vascular endothelial growth factor	6p12	NM_003376	14391	8
NPY2R	Neuropeptide Y receptor Y2	4q31	NM_000910	8447	2
FGF2	Fibroblast growth factor 2	4q26-q27	NM_002006	71528	3
FOS	V-fos FBJ murine osteosarcoma viral oncogene homolog	14q24.3	NM_005252	3383	4
FOSB	FBJ murine osteosarcoma viral oncogene homolog B	19q13.32	NM_006732	7184	4
SOX5	SRY (sex determining region Y)-box 5	12p12.1	NM_152989	1030149	18
SOX6	SRY (sex determining region Y)-box 6	11p15.3	NM_033326	506124	16
SOX9	SRY (sex determining region Y)-box 9	17q24.3-q25.1	NM_000346	5401	3
PTGER4	Prostaglandin E receptor 4 (subtype EP4)	5p13.1	NM_000958	13804	3
CSK	C-src tyrosine kinase	15q23-q25	NM_004383	20790	13
FGF23	Fibroblast growth factor 23	12p13.3	NM_020638	11502	3
FOSL2	Fos-like antigen 2	2p23-p22	NM_005253	21736	4
REL	V-rel reticuloendotheliosis viral oncogene homolog (avian)	2p13-p12	NM_002908	41427	11
RELA	V-rel reticuloendotheliosis viral oncogene homolog A, p65 (avian)	11q13	NM_021975	8559	11
RELB	V-rel reticuloendotheliosis viral oncogene homolog B (avian)	19q13.32	NM_006509	36741	11
NFKB2	Nuclear factor of kappa light polypeptide gene enhancer in B-cells 2 (p49/p100)	10q24	NM_002502	6805	23
ITGAV	Integrin, alpha V (vitronectin receptor, alpha polypeptide, antigen CD51)	2q31-q32	NM_002210	90828	30
JDP2	Jun dimerization protein 2	14q24.3	NM_130469	38320	4
NFATC1	Nuclear factor of activated T-cells, cytoplasmic, calcineurin-dependent 1	18q23	NM_172390	72406	8
PPP3CA	Protein phosphatase 3 (formerly 2B), catalytic subunit, alpha isoform (calcineurin A alpha)	4q24	NM_000944	323767	14
CASR	Calcium sensing receptor	3q21.1	NM_000388	102813	7
ZNF675	Zinc finger protein 675	19p12	AB_209601	33969	4
TRAF6	TNF receptor associated factor 6	11p12	NM_004620	21100	7
CLCN7	Chloride channel 7	16p13.3	NM_001287	29668	25
DBI	Diazepam binding inhibitor (acyl-Coenzyme A binding protein)	2q14.2	NM_020548	5461	5
CTNNB1	Catenin (cadherin-associated protein), beta 1, 88 kDa	3p21	NM_001904	40935	15
TCF4	Transcription factor 4	18q21.2	NM_003199	360475	20
OMD	Osteomodulin	9q22.31	NM_005014	10023	3
DMP1	Dentin matrix acidic phosphoprotein	4q22.1	NM_004407	14049	6
WIF1	WNT inhibitory factor 1	12q14.3	NM_007191	70681	10
MEPE	Matrix extracellular phosphoglycoprotein with ASARM motif (bone)	4q22.1	NM_020203	13807	4
CCR1	Chemokine (C-C motif) receptor 1	3p21.31	NM_001295	6101	2
ATP6V0D2	ATPase, H+ transporting, lysosomal 38 kDa, V0 subunit d2	8q21.3	NM_152565	55316	8
IL15	Interleukin 15	4q31.21	NM_172174	96859	8
VEGFC	Vascular endothelial growth factor C	4q34.3	BC_063685	109205	7
PTK2B	PTK2B protein tyrosine kinase 2 beta	8p21.2	NM_004103	147905	35
WNT9A	Wingless-type MMTV integration site family, member 9A	1q42.13	NM_003395	26903	4
PPARA	Peroxisome proliferative activated receptor, alpha	22q13.31	NM_001001928	93155	8
CAT	Catalase	11p13	NM_001752	33115	13
GPX1	Glutathione peroxidase 1	3p21.3	NM_201397	1181	1
ITGA1	Integrin, alpha 1\	5q11.2	NM_181501	165350	29
MITF	Microphthalmia-associated transcription factor	3p14.2-p14.1	NM_198159	228855	10
PLXNA2	Plexin A2	1q32.2	NM_025179	216898	32
PTGS2	Prostaglandin-endoperoxide synthase 2	1q25.2-q25.3	NM_000963	8588	10
SEMA7A	Semaphorin 7A, GPI membrane anchor	15q22.3-q23	NM_003612	23954	14
DKK1	Dickkopf homolog 1	10q11.2	NM_012242	3063	4
IL3	Interleukin 3 (colony-stimulating factor, multiple)	5q31.1	NM_000588	2550	5
IL13	Interleukin 13	5q31	NM_002188	2937	4

### Statistical analysis

The HapMap database [66] was used to compare the Korean population with other populations. To measure the genetic differentiation between populations, Wright's *F*_*ST *_(the classic measure of population divergence) was calculated from the genotypic data. Haplotypes were suggested using the Partition Ligation-Expectation Maximization (PL-EM) algorithm [67]. We used the KSNP database to analyze LD and haplotype blocks and for tagging the detected SNPs. We defined LD blocks according to the method of LD-based blocking with bootstrapping [68], and haplotype tagging of selected SNPs was accomplished using the Entropy method [69].

## Results

### Identification of SNPs in candidate osteoporosis genes in the Korean population

**Figure 1:**
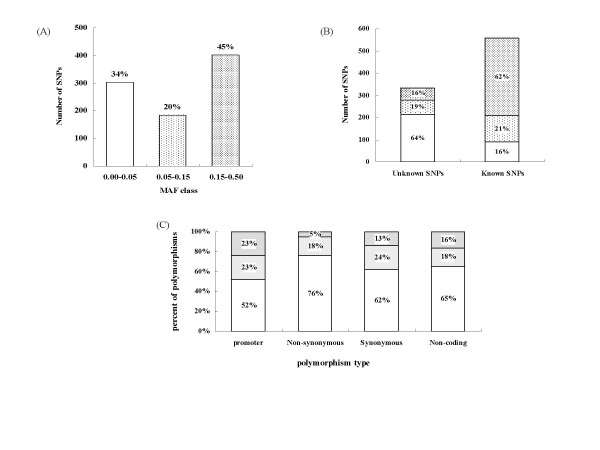
Distribution of the SNPs identified in the 81 candidate osteoporosis genes. (A) Classification of the SNPs into minor allele frequency (MAF) classes. (B) Number of known and unknown SNPs. (C) Distribution of SNPs according to location or type. The percentages in (A), (B), and (C) refer to the percentage of SNPs within each MAF class in the given categories.

We directly sequenced 81 candidate osteoporosis genes including all exons, their intron boundaries, and ~1.5 kb of the 5' flanking region. We identified 942 variants, including 888 SNPs, 43 insertion/deletion polymorphisms, and 11 microsatellite markers (Table 2). Of the 888 SNPs, 118 were located in promoter regions, 21 in 5' untranslated regions (UTRs), 157 in coding regions, 435 in introns, 119 in 3' UTRs and 38 in intergenic regions (Table 2). With regard to the minor allele frequency (MAF), we classified the 888 SNPs into low (MAF < 0.05), intermediate (0.05–0.15), and high (>0.15) frequency classes as described by Cargill et al [70] (Fig. 1A). Of the 888 SNPs, we identified 331 unknown SNPs which were not reported in dbSNP (build 124), and the rest were known (Fig. 1B). Of the 888 SNPs, 401 belonged to the high MAF class, of which 53 (13.2%) were unknown SNPs. In addition, the majority of the low MAF class (70.3%) were also unknown SNPs, suggesting that a large portion of newly identified SNPs exist in a recessive model. Overall, about two-third of the SNPs identified in this study are common in the Korean population (MAF > 0.05). When functionally classified, 76% of the nonsynonymous SNPs (cSNP) belonged to the low MAF class whereas only 52.2% of SNPs in the promoter regions belonged to this class (Fig. 1C). In addition, newly identified SNPs with MAF > 0.15 represented 16% of all the discovered SNPs. In functional aspect, we found some unknown SNPs in the coding region of the genes encoding interleukin 6 signal transducer (*IL6ST*), the androgen receptor (*AR*), and the core-binding factor beta subunit (*CBFB*) which were not reported in dbSNP database. However, there were no SNPs in the coding region of *NFKB2 *in both our dataset and dbSNP, suggesting that they are functionally and evolutionary highly conserved genes.

**Table 2:** Summary of polymorphisms discovered in candidate osteoporosis genes

**Gene**	**IND**		**MIC**		**SNP**															
					
					**Promoter**		**5'UTR**		**Syn**		**Nonsyn**		**3'UTR**		**Intron**		**Intergenic**		**Total**	
	
	^a^T	^b^N	T	N	T	N	T	N	T	N	T	N	T	N	T	N	T	N	T	N
BGLAP					1						1	1							2	1
CALCR	3	2					1				1		4	2	3	1			9	3
IL6ST					1	1	1	1			2	1			5	5			9	8
LGALS3									1	1	3	1			4	1			8	3
PPARG			2	2			1		1						3	2			5	2
PTH									1				1	1	1				3	1
SP7									1										1	0
TGFBI									5	1			2		12	4			19	5
AR									2	2	5	5	1	1	4	4	1	1	13	13
BMP7													1		7	1			8	1
AHSG									2		2				2				6	0
BMP2									1		3	1			1				5	1
BMP4											1				9	5			10	5
BMP6									2	1	1	1	2	1	4	1			9	4
CBFB											1	1	5	5	4	4			10	10
CTSK											1	1			1	1			2	2
DLX5											1	1			1	1			2	2
IBSP					2	2			2		5								9	2
IL1RN					4	1	3		1				1		24		1		34	1
LGALS1					1										4	2			5	2
MSX1			1												2	1			2	1
MSX2	1	1	1										2						2	0
PTHLH	1										1	1	1		2	1			4	2
PTHR1							1	1	1						4				6	1
RUNX1													11	10	3	1			14	11
SPP1					6	5			2				3	1	7	2			18	8
TWIST1	2	2			1	1	1												2	1
FGF2													6	1	2	1	1	1	9	3
FOS	1				1		2		1						1				5	0
FOSB					1	1							3	1	1				5	2
CSK									3	2			3		3	3			9	5
PTGER4					2	1							2	2					4	3
FGF23											2	1							2	1
FOSL2											1	1			2				3	1
ITGAV											1		3	1	14	7			18	8
REL											1	1			2	1			3	2
RELA															2				2	0
RELB			1		1	1							1	1	3	1			5	3
SOX5	1	1							2	2			1	1	2				5	3
SOX6									2	1					8	6			10	7
SOX9													2		1	1			3	1
IL3											1								1	0
IL4					1						1	1			3				5	1
IL13											1		1		1				3	0
NFKB1	3	3			3	2	1	1			2	2			23	5			29	10
VEGF	3	3			1		2								10	3	6	1	19	4
IL10	1	1											3	1	5	2			8	3
NPY2R	1	1	1	1					2		1	1	1	1	10	6			14	8
CAT					2		1		1		2	2			9	2	2		17	4
CEBPB					4	2													4	2
CYP17A1					2		1		3	1	1	1			10	1			17	3
CYP19A1	2	1							2	1	2	2			15	4	2		21	7
GPX1					1								1						2	0
ITGA1	2	2			5	2			2		5	2	13	3	31	3	5	1	61	11
MITF					4	2					1	1	5	3	3	2	2	1	15	9
PLXNA2	1								5	2	8	2			33	16	1	1	47	21
PTGS2	2				4		1		3		2		3		6		3		22	0
SEMA7A					1	1			5	1	1	1	2		6	3			15	6
DKK1					1				1						2	1			4	1
CASR	2	2			2		1	1	2	2	3	1	2		5	1			15	5
CCR1	2	2							1	1	1	1	5		10	5	1		18	7
CLCN7	2	2			10	5			2	1			2	1	12	5	1		27	12
CTNNB1	1	1			1								3	1	3	1			7	2
DBI					11	4			1	1					4	1	1		17	6
DMP1	2	2			6						2	1	2		4				14	1
IL15					2	1							4	1	10	2			16	4
JDP2					2						1		2						5	0
MEPE									1		1	1	1		9	2			12	3
NFATC1					4	1			3		1	1	1	1	9	6			18	9
OMD					4						1	1	1	1					6	2
PPARA	1	1	1	1	2	1			1	1	2	1					6	5	11	8
PPP3CA	1	1	1	1	2								4	3	7	5			13	8
PTK2B					8	4	3	1	10	3	1		1		24	6	3	2	50	16
TCF4	1	1	1	1					1						11	7			12	7
TRAF6	3	3			3	1					1	1	1	1					5	3
VEGFC	1	1			2	2			1	1					3	1			6	4
WIF1	1	1	1	1	4	4							1		3	1			8	5
WNT9A					2				2				3	1	1	1	1		9	2
ATP6V0D2	2	1	1	1	3	1	1	1	1	1			2	1	3		1	1	11	5
ZNF675									1	1	1				2	2			4	3

Total	**43**	35	**11**	8	**118**	46	**21**	6	**81**	27	**76**	40	**119**	47	**435**	151	**38**	14	**888**	331

SNPs, single nucleotide polymorphisms; IND, insertion/deletion polymorphism; MIC, microsatellite; UTR, untranslated region. Syn, Synonymous cSNP, indicates there is no change in the amino acid; Nonsyn, Nonsynonymous cSNP, substitutions in coding regions that result in a different amino acid

^a^T, sum of known SNPs and newly identified SNPs; ^b^N, newly identified SNP in this study

It has been reported that the Japanese SNP database (JSNP) was constructed through the gene-based resquencing method of 24 individuals [71]. Therefore, the newly discovered SNPs for candidate genes of osteoporosis from this study were compared with those in the JSNP database. Of 70 SNPs in the exon region (excluding UTR) with MAF > 0.05 in our data, 28 SNPs were common between our study and the JSNP database. The ratio of the common SNPs to all SNPs from our data and those from JSNP for the selected genes was 28/70 and 28/43, respectively.

### Deviation in Heterozygosity and Genetic diversity

**Figure 2:**
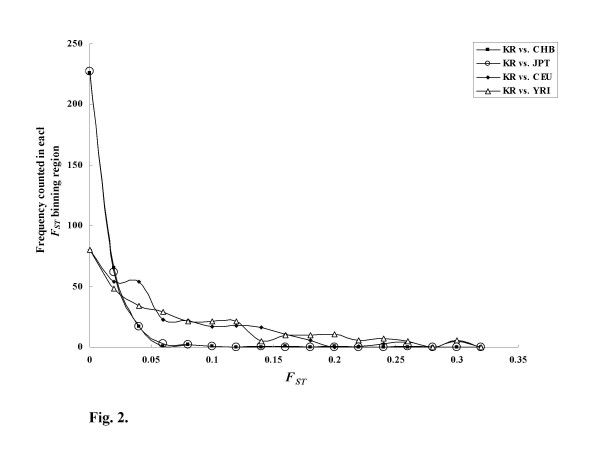
Distribution of *F*_*ST *_among the sub-populations.

**Figure 3:**
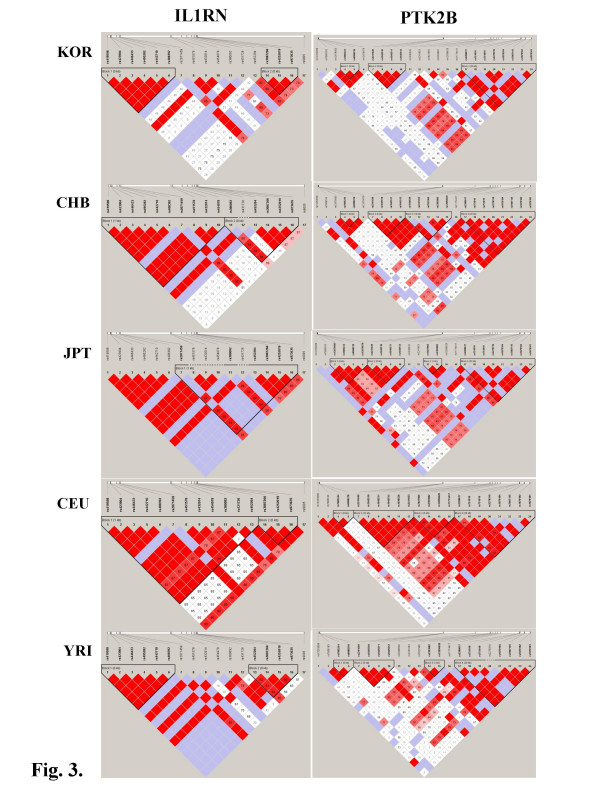
Comparison of LD patterns of PTK2B and IL1RN among the sub-populations.

We used HapMap to compare the allele frequencies of diverse ethnic groups with that of the Korean population [72]. Among the 557 known SNPs detected in this study, 313 were found in HapMap. We thus evaluated genetic differences between Koreans and the diverse populations by measuring the Wright's *F*_*ST *_coefficients using the 313 common SNPs assuming the Hardy-Weinberg principle. *F*_*IS *_is the average deviation in heterozygosity within subpopulations, *F*_*ST *_is the deviation due to subdivision alone, and *F*_*IT *_is the overall deviation in heterozygosity in the total population [73]. The mean values of *F*_*IS*_, *F*_*ST *_and *F*_*IT *_for multiple loci with five subpopulations (KR, CHB, JPT, CEU and YRI) are -0.0121, 0.3366 and 0.3287, respectively, indicating that the SNPs in genes associated with osteoporosis were significantly differentiated among the five subpopulations while the SNPs within the subpopulations were consistent with the Hardy-Weinberg expectations. In addition, the pairwise *F*_*ST *_(s) of KR compared with each of the four subpopulations using the 313 individual SNPs were calculated. The distribution of the pairwise *F*_*ST *_(s) values is plotted in Fig. 2. Interestingly, two distribution patterns were observed that grouped KR-CHB with KR-JPT and KR-CEU with KR-YRI. In addition, the *F*_*ST *_values for KR-CHB and KR-JPT continually decreased to 0.05 whereas those of KR-CEU and KR-YRI continued to 0.2 or more from which point the overall major and minor alleles are reversed, suggesting that there is a large genetic barrier among continental populations. When a threshold (*F*_*ST *_= 0.1 or higher) as the level of significance was applied [74] to our data, 2, 2, 73 and 92 out of 313 SNPs were significantly deviated between KR compared with CHB, JPT, YRI and CEU, respectively. In order to investigate the difference in linkage disequilibrium (LD) patterns between the significantly diverse SNPs in the sub-populations, two highly polymorphic genes (PTK2B and IL1RN) in terms of the number of SNPs per gene were selected and their Haplotype blocks using Haploview [75,76] were plotted against five subpopulations, KR, CHB, JPT, CEU and YRI, as shown in Fig. 3. Interestingly, all five haplotype blocks for each gene were different from one another. Overall, the largest block was found in the CEU population whereas smaller blocks were found in the two genes of the KR and YRI populations. This result implies that determining genetic properties, such as, LD is a powerful method to elucidate the subtle differences in genetic diversity between sub-populations.

In order to determine the genetic diversity between subpopulations, both Nei's standard genetic distance and Latter's *F*_*ST *_distance were also calculated [77,78] and listed in Table 3. Overall, both distance measures agreed with each other in terms of the trend, but overall, Nei's distances were lower than those of Latter's. The genetic distance between the KR and either the CHB (0.012) or the JPT (0.013) subpopulations was very close to each other. On the other hand, the genetic distance of the KR population was closer to the YRI population (0.594) than that of the CEU population (0.646) in these SNPs of selected genes. Therefore, the genetic diversity between KR compared with the other populations for the selected genes also agreed with the *F*_*ST *_analysis result.

**Table 3:** Pairwise genetic distance among five population

	KOR	CHB	JPT	CEU	YRI
KOR		0.01197	0.01331	0.64578	0.59374
CHB	0.05259		0.01207	0.68158	0.58691
JPT	0.05775	0.04840		0.71527	0.60823
CEU	0.85137	0.86316	0.86480		1.00000
YRI	0.83252	0.82958	0.83475	1.00000	

The upper triangle is for Nei's standard genetic distance [19] and the lower triangle is for Latter's *F*_*ST *_distance [20].

## Discussion

In this study, 81 candidate genes of osteoporosis were sequenced to identify common genetic polymorphisms that might alter bone remodeling. In the analysis of differences among ethnic group allele frequencies using the measure of genetic distance, we showed that the Han Chinese and Japanese populations were close to the Korean population. This implies a strong genetic linkage among the Han Chinese, Japanese and Korean populations, which may reflect either a recent common ancestry or high levels of mutual immigration among these groups [79].

The 888 polymorphisms identified in this study were obtained from 24 unrelated individuals. Three hundred and thirty-one (37.3%) variants were newly identified polymorphisms that were not present in the public database examined, whereas 557 (62.7%) of the polymorphisms found by resequencing were already present in the database. Of the 331 variants that were not reported in the database, 64.4% belonged to the low minor allele frequency group (MAF < 0.05) in Koreans and variants, 35.6% were common SNPs in the Korean population. These common SNPs could be useful for further case-control association studies of osteoporosis in Koreans. We identified new SNPs that had low allele frequencies. This may be due to the fact that previous studies used various factors, such as a mixture of populations, or had a relatively smaller sample size, thereby limiting their ability to discover low allele frequency SNPs. Alternatively, as an ethnically homogeneous population, the Korean samples may have allele frequencies that significantly differ from those from mixed samples. Of the 557 variants that were already present in the dbSNP database, only 16.2% had a minor allele frequency lower than 0.05, 21.4% between 0.05 to 0.15 and 62.5% greater than 0.15. Therefore, our resequencing effort provided experimental validation for more than 460 polymorphisms that were already in the database.

In our study, we measured the LD block structure of the candidate genes, excluding cases of one or two SNPs and uncommon SNPs (MAF < 0.05) in each gene, from the limited sample using normalized D' statistics between all pairwise SNP markers with MAF > 0.05 that satisfied the Hardy-Weinberg's equilibrium (p < 0.05). The LD and haplotype results are shown in the KSNP database [65]. A comparison of the haplotype blocks of two highly polymorphic genes (PTK2B and IL1RN) from the KR population with those from the 4 subpopulations in HapMap, showed diverse block patterns (Fig. 3). Therefore, the LD and haplotype information could be valuable resources for ethnicity comparison, tagging SNPs and recombination signals of the osteoporosis-related genes in future studies.

In this study, the nonsynonymous cSNPs tended to have a larger proportion of low allele frequencies compared with the synonymous cSNPs, the noncoding SNPs, and the promoter SNPs. This trend is consistent with a selection pressure against SNPs that cause amino acid changes [80]. In contrast, the promoter regions, which had a wide range of allele frequencies overall, had more SNPs with high allele frequency compared with the other regions. These results indicate that the promoter variants found in this study might be utilized as genetic determinants for future studies [81]. The several million human SNPs reported in the HapMap international project will likely prove useful for association studies; however SNPs located close to functionally important genes are more valuable as markers than random genomic SNPs. Moreover, SNPs located in the coding or promoter regions have the added benefit of potentially causing the genetic variation that directly contributes to disease. Therefore, additional resequencing efforts are still needed for comprehensive studies of osteoporosis candidate genes across ethnic groups as such data should prove important for future association studies of osteoporosis.

## Conclusion

We directly resequenced 81 candidate osteoporosis genes and identified 942 variants including 888 SNPs, 43 insertion/deletion polymorphisms, and 11 microsatellite markers. Of the 888 SNPs, 331 SNPs have not been previously identified and 557 SNPs were already reported in the dbSNP database, of which more than 460 were validated by our resequencing effort.

Statistical analysis of deviation in heterozygosity with the HapMap data depicted that compared with SNPs in Koreans, 1%(or less) of SNPs in Japanese and Chinese and 20% of those in Caucasian and African were significantly differentiated from the Hardy-Weinberg expectations. In addition, the analysis of genetic diversity between Korean and the other four populations showed that the order of the closest neighbor (in terms of genetic distance) is Han Chinese, Japanese, African and Caucasian. In general, we didn't find any significant differences among three sub-populations from KR, CHB and JPT, but these Asian populations, CEU and YRI were significantly different in both the *F*_*ST *_and genetic diversity results in selected genes. Nevertheless, analysis using genetic properties, such as LD and haplotype patterns showed that all-sub populations were substantially different.

Overall, through the resequencing of 81 osteoporosis candidate genes, 118 unknown SNPs with MAF > 0.05 were discovered in a Korean population. In addition, our newly discovered SNPs were compared with those in HapMap to elucidate diversity and deviation in heterozygosity, resulting in strong genetic linkages between the Han Chinese, Japanese and Korean populations. This result may reflect either a recent common ancestry or high levels of mutual immigration among these groups. Yet, using a genetic property, such as LD patterns, is a powerful method to elucidate the subtle differences between the Korean, Chinese and Japanese populations. Our results could aid in the design of case-controlled and population stratification studies in the Korean population.

## Competing interests

The author(s) declare that they have no competing interests.

## Authors' contributions

KS and GS performed genome sequence analysis, produced the results and drafted the manuscript. JY, HJ, MH, HD and JH contributed to the preparation of samples and sequence alignment in the study. KJ and HS provided technical assistance. JS performed the statistical analysis with *F*_*ST *_and calculated the genetic distances between the sub-populations and revised the manuscript. JM, EK and TH selected the candidate genes involved in bone resorption using a microarray experiment and revised the manuscript. BS, KC and HL have been involved in critically revising the manuscript for important intellectual content. JYL and SY contributed to the conception of the study and participated in the interpretation of the results and revision of the manuscript. All authors read and approved the final manuscript.

## Pre-publication history

The pre-publication history for this paper can be accessed here:


